# Correlation between adipokines and carotid intima media thickness in a group of obese Romanian children: is small for gestational age status an independent factor for cardiovascular risk?

**DOI:** 10.1590/2359-3997000000201

**Published:** 2016-08-31

**Authors:** Ramona Stroescu, Teofana Bizerea, Gabriela Doroş, Monica Marazan, Maria Lesovici, Otilia Mãrginean

**Affiliations:** 1 “Louis Turcanu” Emergency Hospital for Children Timişoara România “Louis Turcanu” Emergency Hospital for Children, Timişoara, România; 2 “Victor Babes” University of Medicine and Pharmacy Timişoara România “Victor Babes” University of Medicine and Pharmacy, Timişoara, România

## Abstract

**Objective:**

To investigate the relationship between markers of adiposity and common carotid artery (CIMT) in obese children born small for gestational age (SGA) versus appropriate for gestational age (AGA), to establish cut-off values for CIMT in obese pediatric populations.

**Subjects and methods:**

A cross-sectional study was carried out over a 1-year period (Jul 2013 – June 2014). We analyzed 122 obese patients aged 4-20 (mean age 14.9 ± 2.28). Twenty-six patients were born SGA. CIMT was measured in all the patients. Using ROC curve, cut-off values were obtained for both groups.

**Results:**

We demonstrated a correlation between CIMT and adiponectin, leptin and high sensitivity C-reactive protein (hsCRP) (r = -0.25, r = 0.279, r = 0.498) in obese children. CIMT in obese children born SGA were significantly increased as compared with obese children born AGA of similar age, sex and body mass index (BMI) (p = 0.0035). A CIMT cut off value of 0.049 cm has been obtained with a high sensitivity and specificity.

**Conclusion:**

CIMT is a well-known marker of subclinical atherosclerosis and its measurement is a noninvasive and inexpensive method of detecting subclinical atherosclerosis. Being born SGA increases the atherogenic risk. Obese children with CIMT above 0.049 cm should be screened for metabolic syndrome (MetS).

## INTRODUCTION

CIMT provides an index of atherosclerosis in other vascular regions ( 1 - 5 ) and has been shown to be associated with most risk factors for atherosclerosis ( 6 - 8 ). Increased values of CIMT determined by B-mode ultrasound have been shown to be directly associated with an elevated risk of myocardial infarction and stroke in older adults without a previous history of cardiovascular disease ( 9 ). Thus, CIMT has been proposed as a risk factor that may be included in the algorithms for cardiovascular risk assessment ( 9 ).

The pathogenesis of atherosclerosis as identified by the Pathobiological Determinants of Atherosclerosis in Youth trial has endothelial dysfunction as its first step; an accumulation of modified lipoproteins cause a thickening of the intima; macrophages attach to the lipoproteins, resulting in the formation of foam cells, which accumulate in the intima forming a fatty streak; this step is followed by proliferation of smooth muscle cells, developing a fibrous area ( 10 ). Atherosclerotic modifications of blood vessels start in childhood, although clinical manifestations of atherosclerosis mainly occur in adults. In children and adolescents, atherosclerosis can present itself as a diffuse thickening of the intima-media space rather than the common structure of fatty streaks and fibrous capsules ( 11 ).

CIMT is commonly used as a marker of atherosclerosis; a predictive association with cardiovascular disease has been established. In adults, CIMT > 0.9 mm has been shown as a marker of cardiovascular risk caused by atherosclerosis ( 12 ). In children, there are few data regarding reference values for CIMT.

Adipose tissue is an important endocrine organ that produces interleukin-6 (IL−6), tumor necrosis factor alpha (TNF-α), plasminogen activator inhibitor-1 and adipokines (leptin and adiponectin). Adipokines control energy homeostasis and are involved in metabolic, endocrine and immunological processes.

Leptin is a hormone closely linked to adipose tissue and its levels are directly influenced by the amount of body fat mass and size of individual adipocytes; through its actions on the medial and lateral hypothalamus, it controls food intake and energy expenditure. In obesity, however, it was observed that high levels of leptin do not reduce appetite; a possible explanation may be a state of leptin resistance. It also has a pro-inflammatory action, stimulating the secretion of IL−6 and TNF-α ( 13 , 14 ).

Adiponectin, as opposed to leptin, is a hormone inversely correlated with body fat mass. Adiponectin is the most abundant of the adipokines and is an insulin-sensitizing hormone with anti-inflammatory, anti-atherogenic and anti-diabetic properties ( 15 , 16 ).

High sensitivity C-reactive protein (hsCRP) is a biomarker of the low grade chronic inflammation associated with atherosclerosis. Likewise, CRP is one of the most important inflammatory markers in obesity. Several studies showed that hsCRP is a strong independent predictor of cardiovascular risk. It has a role at different phases of atherosclerosis ( 17 ). Adults with hsCRP above 0.3 mg/dL have twice the risk of atherosclerosis compared to those with low levels (< 0.1 mg/dL) ( 18 , 19 ). There are also studies indicating the utility of hsCRP in assessing cardiovascular risk in obese children ( 20 ).

Chronic inflammation in obesity leads to endothelial dysfunction, followed by further atherosclerotic modifications of blood vessels.

Children and adolescents with risk factors such as obesity, dyslipidemia, high blood pressure and impaired glucose metabolism are at risk for atherosclerosis in adulthood ( 21 - 23 ). Childhood obesity correlates with early onset of cardiovascular disease in adults. Children born small for gestational age (SGA) have a high risk of developing MetS with all its components.

This study aimed to investigate the relationship between markers of adiposity, like leptin, adiponectin, hsCRP and CIMT in obese children, to compare values of CIMT and markers of adiposity between children born SGA and AGA, and to establish cut off values for CIMT in obese pediatric populations.

## SUBJECTS AND METHODS

A cross-sectional study was conducted over a period of 1 year, between July 2013 and June 2014, on cases of obesity in children diagnosed at the Emergency Hospital for Children “Louis Ţurcanu” Timişoara, in the departments of Diabetes and Nutritional Diseases, Endocrinology and Cardiology.

Subjects were considered obese on the basis of age-specific BMI reference guidelines from Centers for Disease Control and Prevention Child Growth Standards 2000 (above 95^th^ percentile) ( 24 ). When defining SGA, growth nomograms and charts proposed by Niklasson and cols. ( 25 ) were used; subjects whose birth weight was more than 2 standard deviations (SD) below the average for gestational age were considered SGA; subjects with birth weight within normal range for gestational age were defined as AGA.

CIMT was measured by B-mode ultrasound using a 10-MHz linear transducer (General Electric). The subjects were examined in the supine position with the neck extended and the probe in the antero-lateral position. All measurements of CIMT were made in the longitudinal plane at the point of maximum thickness on the far wall of the right common carotid artery along a 1 cm section of the artery proximal to the carotid bulb. CIMT was defined as the distance between the intima-blood interface and the adventitia-media junction. After freezing the image, measurements were made using electronic calipers. The maximal thicknesses of the intima-media width were measured to give three readings and the mean value was used for statistical purposes.

hsCRP was determined by immunonephelometry from serum samples and processed in a BN ProSpec^®^ system (Siemens Healthcare Diagnostics Inc.) (undetected if < 0.02 mg/dL).

Quantitative measurements of serum leptin and adiponectin levels were performed using commercially available enzyme-linked immunosorbent assay kits (Antisel and Diamedix).

Exclusion criteria were evidenced for endocrine or syndromal disorders of obesity, systemic disease or acute illness.

We analyzed 122 patients diagnosed with obesity: 96 patients born AGA and 26 patients born SGA. Both groups were matched for age, sex and BMI. CIMT was also measured in members of a control group of 42 nonobese patients matched for age, sex and birth weight with the obese group (SGA+AGA).

The data are expressed as means ± standard deviation or as frequencies. Statistical analysis was performed with MedCalc. Pearson’s correlation was used to establish correlations between the parameters in the study group. A p < 0.05 was considered statistically significant. ROC curve was used for determining the optimal cut-off value for CIMT in obese children.

Consent was obtained from the parents and the Ethical Committee of the hospital.

## RESULTS

CIMT values measured in the nonobese group were significantly lower than those in the obese group (0.035 cm vs 0.046 cm, p < 0.01) as shown in Table 1 .

**Table 1:** The anthropometric characteristics and CIMT values of the nonobese group and of the obese group

Total number	Obese group (SGA+AGA) 122					Non obese group 42				P value
	
	Mean	SD			Range	Mean	SD	Range		
Age (years)	14.9	2.3			4-20	14.5	2.48	4-18		0.68
Birth weight (grams)	3231.72	610			970-4300	3032.87	388.31	2350-3800		0.57
Sex (%)										0.68
Male		40.3%					36.5%			
Female		59.7%					63.5%			
Antropometric data BMI (kg/m^2^)	32.8		7.27		19-54.5	16.09	3.31		7.43-21.85	<0.01
CIMT (cm)	0.046		0.01	0.03-0.09		0.035	0.005		0.025-0.05	<0.01

BMI: body mass index; CIMT: carotid intima media thickness.

The anthropometric and metabolic characteristics and CIMT values of the obese group are shown in Table 2 .

**Table 2:** Anthropometric and metabolic characteristics and CIMT values of AGA versus SGA group

Total number	Obese SGA-group I 26					Obese AGA-group II 96				P value
	
	Mean	SD			Range	Mean	SD	Range		
Age (years)	14.41	3.3			5-17	15.05	2.28	4-20		0.22
Birth weight (grams)	2550	403.5			970-2860	3446.25	461.4	2400-4300		< 0.01
Gestational age (weeks)	38	2.9			30-41	39.368	1.14902	34-41		0.025
Sex (%)										
Male		42.3%					36.5%			
Female		57.7%					63.5%			0.78
Residence urban/rural		57%/43%					60%/40%			0.79
Antropometric data BMI (kg/m^2^)	29.6		8.13		19-54.5	30.6	6.3		17-47	0.5
CIMT (cm)	0.057		0.008	0.04-0.09		0.043	0.008		0.03-0.07	< 0.01
Biological data Leptin (ng/mL)	29.5		14.23	15.2-43.7		26	13.08		12.9-39.1	0.036
Adiponectin (ug/mL)	16.4		2.27	1-48		19.9	2.34		2.4-64	0.04
hsCRP (mg/dL)	0.9		0.24	0.6-1.1		0.7	0.32		0.39-1	0.017

BMI: body mass index; CIMT: carotid intima media thickness; hsCRP: high sensitive C reactive protein.

**Figure 1:**
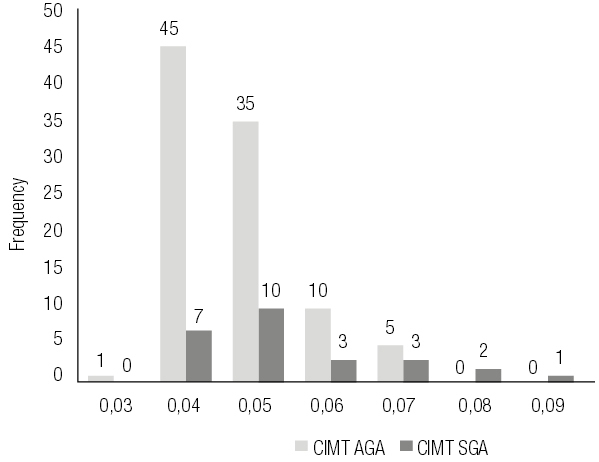
CIMT histogram in SGA and AGA group.

The SGA group had significantly higher levels of leptin and hsCRP (p = 0.036, p = 0.017, respectively) and lower levels of adiponectin (p = 0.04). CIMT was significantly increased in the SGA group (mean 0.057 cm vs 0.043 cm) ( Figure 1 ); there were significant differences between the two groups (p = 0.0035).

### The relationship between CIMT and adypokines

We further aimed to determine correlations between CIMT and the other variables.

CIMT was directly weakly correlated with leptin (r = 0.279; p < 0.05) and strongly with hsCRP (r = 0.498; p = 0.01), and inversely and weakly correlated with adiponectin (r = -0.25; p = 0.04) ( Table 3 ).

**Table 3:** Correlation between CIMT and other variables

	Leptin	Adiponectin	hsCRP
CIMT Pearson’s correlation	0.279	-0.25	0.498
p	0.01	0.04	0.031

hs-CRP: high-sensitivity C-reactive protein.

### Establishing cut-off values using ROC curve in obese children

**Figure 2:**
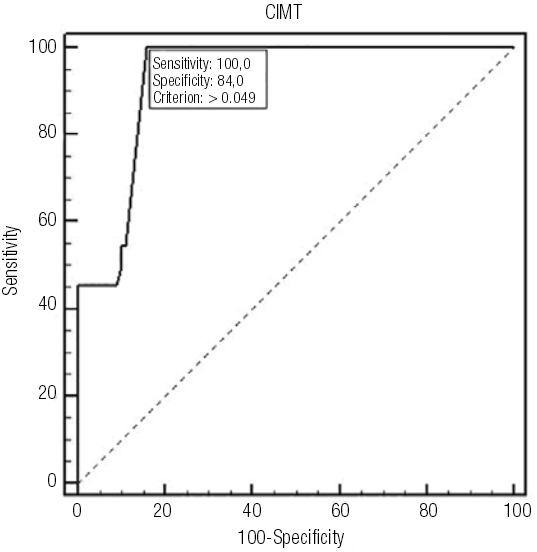
ROC curve SGA group.

**Figure 3:**
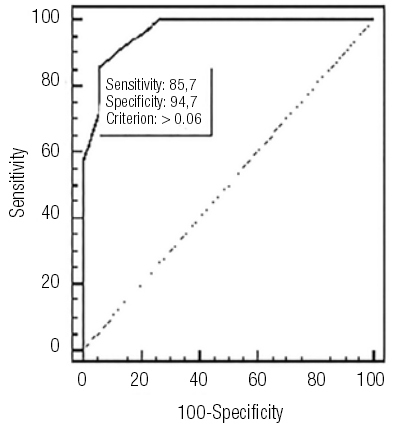
ROC curve SGA+AGA groups.

We have determined cut-off values for the SGA group using ROC curve ( Figure 2 ). The limited size of the SGA sample ( 26 patients) compelled us to establish a cut-off value using CIMT measurements from both groups ( Figure 3 ). This cut-off value of CIMT acts as a designated limit; higher values indicate an increased risk of developing MetS.

Using ROC curve, we obtained for the obese group a cut-off CIMT value of 0.049 cm with a sensitivity of 100% and a specificity of 84%.

## DISCUSSION

Previous reports indicate that the presence of obesity in childhood is associated with increased adult CIMT ( 22 , 23 ). CIMT is a well-known marker of subclinical atherosclerosis and it can also indicate future cardio-cerebrovascular disease ( 26 - 28 ).

In a recent study, we demonstrated a relationship between CIMT, MetS and its components in obese children ( 29 ).

Obesity is a chronic inflammatory disorder in which leptin, adiponectin and CRP play an important role ( 30 ). In this study, a correlation between CIMT adiponectin, leptin and hsCRP was observed. In healthy individuals, adiponectin has anti-atherogenic properties and studies have shown that, in adults, a low level of adiponectin correlates with coronary lesions and is an independent risk factor for the progression of type 2 diabetes ( 31 , 32 ). Previous research suggests that elevated leptin levels are involved in cardio-cerebrovascular disorders such as myocardial infarction and stroke ( 33 ). Leptin induces smooth muscle cell hypertrophy and proliferation through endothelin-1 and angiotensin II dependent mechanisms ( 34 ). Angiotensin II increases the production of reactive oxygen species and induces the release of the vasoconstrictor endothelin-1 ( 35 , 36 ). Leptin also stimulates the secretion of inflammatory markers such as CRP, TNF-α and IL-6, which are directly involved in the development of endothelial dysfunction and atherosclerosis ( 37 , 38 ).

Winer and cols. established that low levels of adiponectin are associated with higher CRP levels ( 39 ). Also, a direct correlation between CIMT and leptin was demonstrated in obese individuals ( 40 ). The weak correlation between CIMT, adiponectin and leptin detected in our study was an indicator of further cumulative risk factors leading to changes in CIMT values. A stronger correlation has been observed between CIMT and hsCRP, suggesting an inflammatory process in obesity. Endothelial dysfunction is considered the earliest step in the atherosclerotic process and precedes any morphological changes in the walls of blood vessels.

The catch-up growth in children born SGA has been associated with a high risk of developing MetS with all its components: obesity, impaired glucose tolerance, insulin resistance with subsequent development of diabetes, arterial hypertension and dyslipidemia. There is also a risk of developing adrenal and reproductive disorders ( 41 ). These changes have been related to intrauterine life environments and linked to epigenetic fetal programming.

Visentin and cols., in a study examining relationships between levels of adipokines (adiponectin and leptin), inflammatory markers (CRP, TNF-a and IL-6) and vascular remodeling in pregnancies with intrauterine growth restriction, found higher CIMT values, high serum concentrations of proinflamatory cytokines (leptin, hsCRP, TNF-a and IL-6) and low levels of adiponectin in fetuses ( 42 ).

In our study, there was a significant statistical difference between AGA and SGA groups regarding CIMT, adiponectin, leptin and hsCRP as shown in Table 1 .

Reference values for CIMT in adults are 0.04-0.07 cm ( 43 ). An increase in CIMT with each decade of age (0.066 cm/decade) was observed, with CIMT values as high as 0.0733 cm in the 70-79 years group ( 44 ). An increase of 0.1 mm in CIMT is known to raise the risk of myocardial infarction by 11% ( 45 ).

In children, there are very few studies on CIMT; normal CIMT is considered to be 0.04 cm ( 46 ). A study assessing CIMT in children aged 6-14 found a median CIMT of 0.048 cm in nonobese children and 0.055 cm in obese children ( 47 ). Another study, which enrolled 128 patients aged 6-18 years, showed a CIMT of 0.043 cm in nonobese children versus 0.051 cm in obese children ( 48 ). In our control group – the nonobese group – we obtained a median CIMT value as low as 0.035 cm. A significant difference from previous results can be clearly observed. An explanation might be the number of adolescents enrolled in the study group; as we know, CIMT value increases with age.

We found little literature data regarding reference values for CIMT in obese children; with this study we achieved threshold values of CIMT maintaining high sensitivity and specificity. For both groups we obtained a cut-off value of 0.049 cm, with a sensitivity of 100% and a specificity of 84%.

It is our belief that these cut-off CIMT values for assessing metabolic risk are “pilot” results. In order to refine these values and offer a reliable tool for determining cardiovascular risk in obese children, further extensive population studies are recommended.

### Limitation

Data from our small clinical samples and the limited number may not be representative for general populations. CIMT may also be influenced by other risk factors that have not been tested in our study.

## CONCLUSION

CIMT is a well-known marker of subclinical atherosclerosis and its measurement is a noninvasive and inexpensive method of detecting subclinical atherosclerosis; it is in relationship to adiponectin, leptin and hsCRP. Being born SGA increases the atherogenic risk. Obese children having CIMT > 0.049 cm should be tested for MetS. Further population studies that look into CIMT values in obese and nonobese children are necessary.
